# *Pm*AP2-β depletion enhanced activation of the Toll signaling pathway during yellow head virus infection in the black tiger shrimp *Penaeus monodon*

**DOI:** 10.1038/s41598-021-89922-w

**Published:** 2021-05-18

**Authors:** Thapanan Jatuyosporn, Pasunee Laohawutthichai, Premruethai Supungul, Rogerio R. Sotelo-Mundo, Adrian Ochoa-Leyva, Anchalee Tassanakajon, Kuakarun Krusong

**Affiliations:** 1 Structural and Computational Biology Research Unit, Department of Biochemistry, Faculty of Science, Chulalongkorn University, Bangkok, 10330 Thailand; 2 Center of Excellence for Molecular Biology and Genomics of Shrimp, Department of Biochemistry, Faculty of Science, Chulalongkorn University, Bangkok, 10330 Thailand; 3 National Center for Genetic Engineering and Biotechnology (BIOTEC), National Science and Technology Development Agency (NSTDA), Pathumthani, 12120 Thailand; 4 Laboratorio de Estructura Biomolecular, Centro de Investigación en Alimentación Y Desarrollo, A.C. (CIAD), Carretera Gustavo Enrique Astiazaran Rosas No. 46, 83304 Hermosillo, Sonora Mexico; 5 Departamentos de Microbiología Molecular, Universidad Nacional Autónoma de México (UNAM), Avenida Universidad 2001, Colonia Chamilpa, 62210 Cuernavaca, Mexico

**Keywords:** Innate immunity, Marine biology

## Abstract

Yellow head virus (YHV) is a pathogen which causes high mortality in penaeid shrimp. Previous studies suggested that YHV enters shrimp cells via clathrin-mediated endocytosis. This research investigated the roles of clathrin adaptor protein 2 subunit β (AP-2β) from *Penaeus monodon* during YHV infection. *Pm*AP2-β was continuously up-regulated more than twofold during 6–36 hpi. Suppression of *Pm*AP2-β significantly reduced YHV copy numbers and delayed shrimp mortality. Quantitative RT-PCR revealed that knockdown of *Pm*AP2-β significantly enhanced the expression level of *Pm*Spätzle, a signaling ligand in the Toll pathway, by 30-fold at 6 and 12 hpi. Moreover, the expression levels of gene components in the Imd and JAK/STAT signaling pathways under the suppression of *Pm*AP2-β during YHV infection were also investigated. Interestingly, anti-lipopolysaccharide factor isoform 3 (ALF*Pm*3) was up-regulated by 40-fold in *Pm*AP2-β knockdown shrimp upon YHV infection. In addition, silencing of *Pm*AP2-β dramatically enhanced crustin*Pm*1 expression in YHV-infected shrimp. Knockdown of ALF*Pm*3 and crustin*Pm*1 significantly reduced shrimp survival rate. Taken together, this work suggested that *Pm*AP2-β-deficiency promoted the Toll pathway signalings, resulting in elevated levels of ALF*Pm*3 and crustin*Pm*1, the crucial antimicrobial peptides in defence against YHV.

## Introduction

Yellow head virus (YHV) is a lethal positive-sense single-stranded RNA virus with a spike envelope. YHV widely infects penaeid shrimps, including *Euphausia superba*, *Litopenaeus setiferus*, *P. merguiensis*, *Metapenaeus ensis*, *L. vannamei*, *P. stylirostris*, *P. setiferus*, *P. aztecus* and *P. duorarum*^1–3^. YHV entry via the clathrin-mediated endocytosis has been identified by endocytosis inhibition and by silencing of the clathrin coated assembly protein 17 (AP17), a σ2 subunit of the adaptor protein complex, and clathrin heavy chain^4,5^. Suppression of *Pm*Rab7, a transportation protein involving in late endosome trafficking, resulted in a decrease in YHV^6^.

In general, clathrin-mediated endocytosis is a well-characterized endocytic mechanism for uptaking nutrients, pathogens antigens, growth factors and receptors. Initiation of the clathrin-mediated endocytosis requires the accumulation of phosphatidylinositol-4,5-bisphosphate (PIP2) and clathrin assembly protein 2 (AP2) complex at the plasma membrane. AP2 consists of 4 subunits, including β2, α, µ2 and σ2. In *P. monodon*, the β2, µ2 and σ2 subunits of AP-2 have been characterized^4,7^.

AP2 complex first binds to the cytoplasmic tail of the ligand-receptor complex and recruits other accessory proteins such as clathrin, epsin and β-arrestin to form clathrin-coated pits, which are then pitched off from the plasma membrane by GTPase dynamin^8^. The endocytic vesicle then fuses with the early endosome from where the ligand-receptor is sorted by either for recycling via Rab4- or Rab11-dependent pathway or for degradation in lysosomes via Rab7^9^. Several viruses, including semliki forest virus^10^, vesicular stomatitis virus^11^, influenza A virus^12^, Foot- and mouth disease virus^13^ and hepatitis C^14^, hijack the clathrin-mediated endocytosis to enter host cells. Previously, the white spot syndrome virus (WSSV), the most devastating pathogen in shrimp, was also reported to invade host cells via clathrin-dependent endocytic route^7,15,16^.

Clathrin-mediated endocytosis is responsible for transporting a wide variety of cargoes from the plasma membrane into the cell. This process does not only maintain membrane compositions but also controls cell-signaling pathways. The internalized ligand-receptor complex remains signal transduction as they are located at plasma membrane^17^. Intervention of endocytosis may disrupt intracellular signaling networks, leading to malfunctioning in many cellular processes such as cell development, migration and neuroplasticity^18,19^. In *Drosophila*, endosomal entry regulates Notch receptor activation^20^ and the endocytic mechanism also controls the JAK/STAT (Janus tyrosine kinase/Signal transducer and activator of transcription) signaling^21^. In HeLaM cells, clathrin-mediated endocytosis of type-I interferon (IFN-α/β) receptor (IFNAR) is required for the activation of JAK/STAT signaling and the activities of type-I IFNs^22^. Clathrin controls Wnt/β-catenin signaling by manipulating exocytosis of transmembrane proteins such as cadherins and Wnt co-receptors^23^. Lipopolysaccharide (LPS) receptor is mediated by clathrin and colocalized with the Toll-like receptor, TLR4, on early/sorting endosomes^24^. The disruption of endocytosis and endosomal sorting results in increased LPS signaling. In addition, the impairment of clathrin internalization enhances expression of lymphotoxin β receptor (LTβR) and activation of canonical NF-κB signaling^25^. These evidences suggest that clathrin-dependent endocytosis could regulate several signaling pathways.

In shrimp, antimicrobial peptides play an important role in defence against viral and bacterial infections. The expression of antimicrobial peptides was controlled by different signaling pathways. The Toll and Immune Deficiency (Imd) signaling pathways are one of the first lines of shrimp innate immunity. Previously, ALF*Pm*3 was reported to have been governed by the Toll and the Imd pathways^26^. Crustin*Pm*1 was regulated by the Toll signaling pathway while crustin*Pm*7 was mediated through both Toll and Imd pathways^27^. Expression of PEN3 was under the regulation of both Toll and Imd pathways, while PEN5 was controlled by the Imd^28^.

In this study, RNA interference techniques, immunofluorescence confocal microscopy, transmission electron microscopy (TEM) and mortality study were employed to investigate the roles of *Pm*AP2-β during YHV infection. The transcription levels of genes in the Toll, Imd and JAK/STAT signaling pathways and other immune response genes were examined under the suppression of *Pm*AP2-β during YHV challenge. This work reveals the roles of clathrin-mediated endocytosis during YHV infection.

## Results

### Influence of *Pm*AP2-β during YHV infection

**Figure 1:**
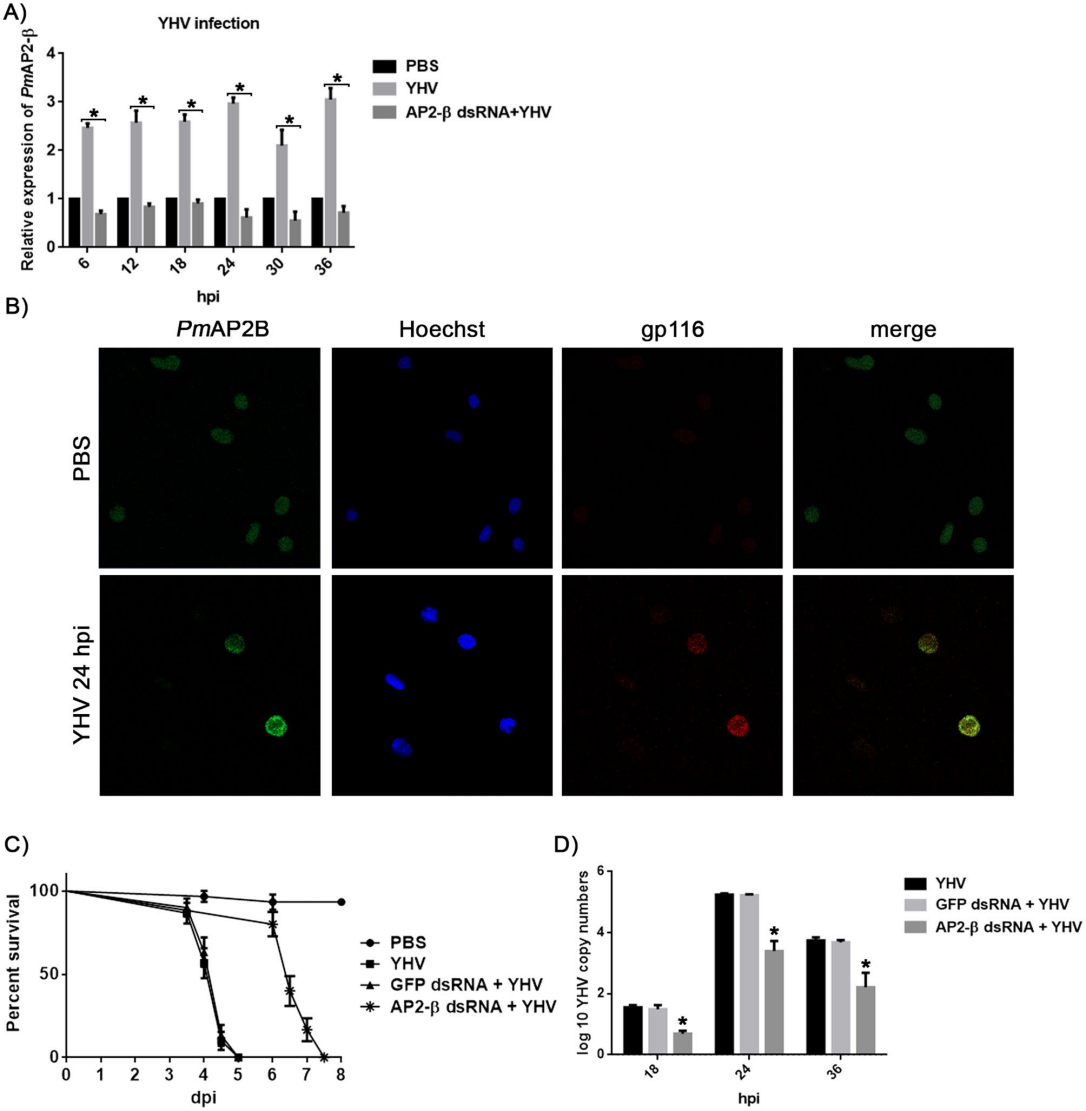
Expression of *Pm*AP2-β and effect of *Pm*AP2-β silencing on YHV infection. (**A**) The relative transcription levels of *Pm*AP2-β during YHV infection. The mRNA expression levels of *Pm*AP2-β were analyzed by Quantitative Real-time RT-PCR. The experiment was carried out in triplicate. (**B**) Expressions of *Pm*AP2-β (green) and gp116 (red) in the hemocytes of unchallenged and YHV-challenged shrimp were observed by confocal laser scanning microscopy. Secondary antibodies conjugated with Alexa Fluor 488 (green) and Alexa Fluor 568 (red) were used to probe anti-AP2-β and gp116 antibodies, respectively, while nuclei were stained in blue. (**C**) Effect of *Pm*AP2-β silencing on cumulative mortality caused by YHV. Shrimp were injected with either 150 mM NaCl or 10 µg GFP dsRNA per 1 g of shrimp or 10 µg *Pm*AP2-β dsRNA per 1 g of shrimp at 24 h prior to YHV challenge. The cumulative mortality was recorded every 12 h after YHV injection. Each group contains 10 shrimps. The experiment was carried out in triplicate. (**D**) Determination of YHV copy number in *Pm*AP2-β knockdown shrimp. Shrimp were divided into three groups and injected with either 150 mM NaCl, GFP dsRNA (10 µg/g shrimp), *Pm*AP2-β dsRNA (10 µg/g shrimp). Shrimp hemocytes were collected at 6, 12, 18, 24, 30 and 36 h after YHV injection for YHV copy number determination. The data are shown as the mean ± standard deviation. An asterisk represents significant differences from control group (*p* < 0.05). The experiment was carried out in triplicate.

To investigate the function of *Pm*AP2-β during YHV infection, shrimp hemocytes were collected at different timepoints after YHV injection to measure the transcription level of *Pm*AP2-β. Based on quantitative RT-PCR analysis, *Pm*AP2-β was constantly up-regulated more than twofold in all observed timepoints (Fig. 1A). Immunofluorescence confocal microscopy confirmed that *Pm*AP2-β was highly expressed at protein level upon YHV infection (Fig. 1B). Silencing of *Pm*AP2-β delayed the cumulative mortality caused by YHV (Fig. 1C) and also reduced YHV copy numbers (Fig. 1D). This suggested that *Pm*AP2-β knockdown interfered with YHV propagation.

### Localization of *Pm*AP2-β during YHV infection

**Figure 2:**
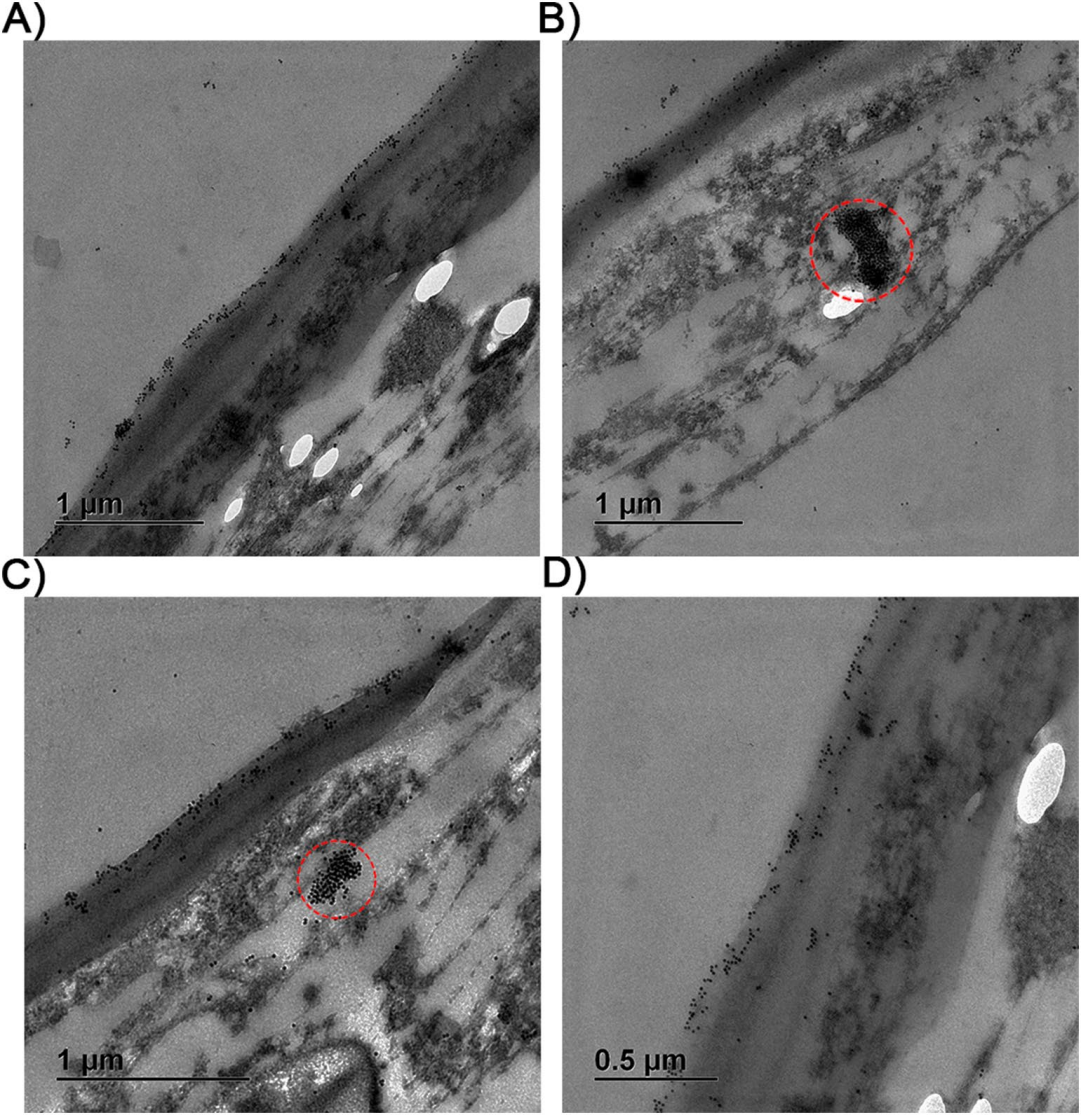
Visualization of *Pm*AP2-β in YHV-infected hemocytes cell by TEM. *Pm*AP-2β was detected by a 10 nm gold-conjugated anti-AP-2β antibody. Red circles show clusters of *Pm*AP2-β form as a sac.

*Pm*AP2-β was probed by a 10-nm gold particle conjugated with *Pm*AP2-β antibody in order to visualize *Pm*AP2-β during YHV infection by transmission electron microscope (TEM). As shown in Fig. 2A–D, *Pm*AP2-β was accumulating around the plasma membrane of YHV-infected shrimp hemocytes. In addition, clusters of *Pm*AP2-β, resembling a sac, were also observed in Fig. 2B,C. Presumably, these *Pm*AP2-β clusters may contain YHV inside.

### Effect of *Pm*AP2-β silencing on the Toll, the Imd and the JAK/STAT signaling pathways during YHV infection

**Figure 3:**
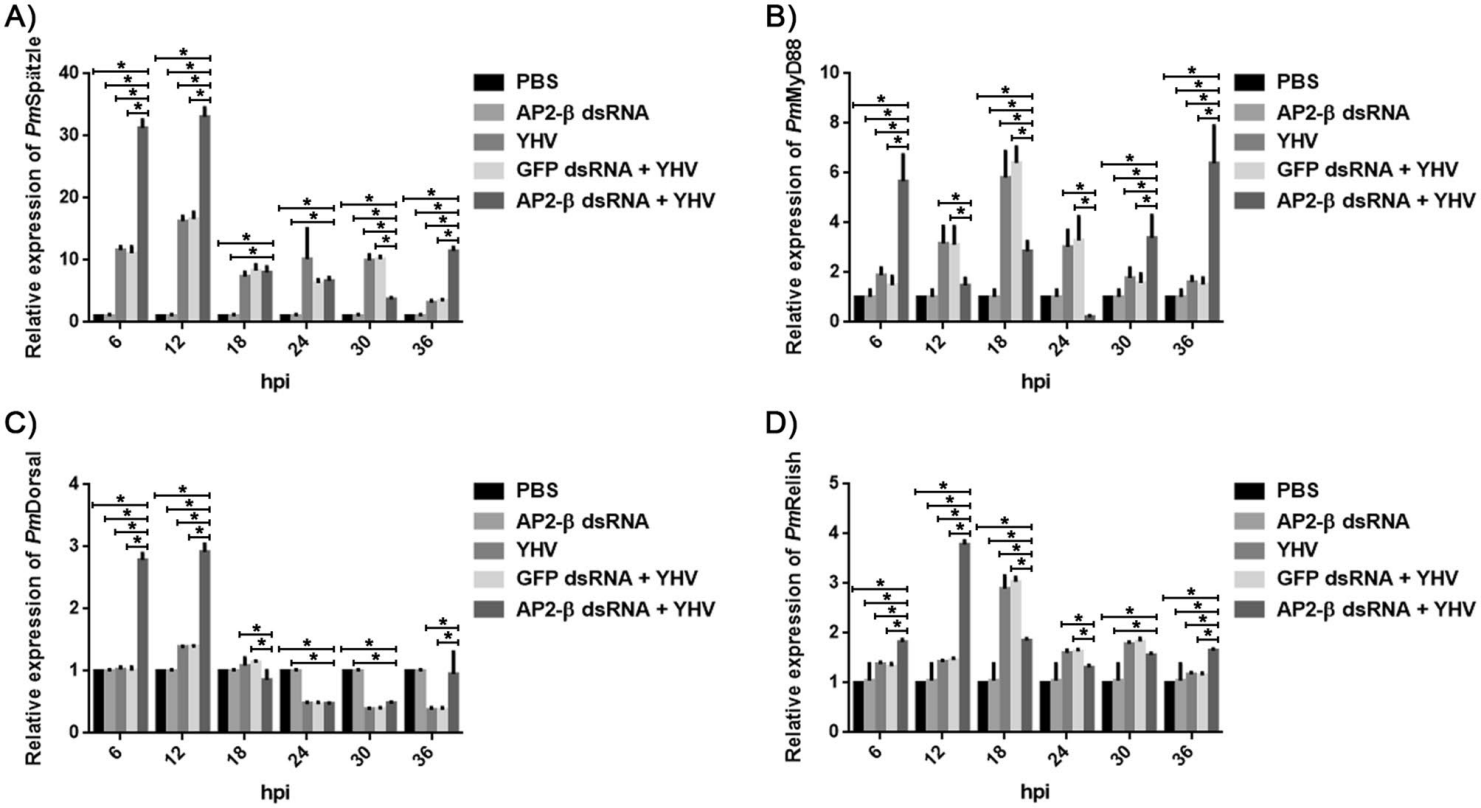
Effect of *Pm*AP2-β silencing on the Toll and the Imd signaling pathways during YHV infection. Shrimp were double injected with either 150 mM NaCl, GFP dsRNA (10 µg/g shrimp) and *Pm*AP2-β dsRNA (10 µg/g shrimp). After YHV injection, shrimp hemocytes were collected at 6, 12, 18, 24, 30, 36 h for qRT-PCR analysis of the transcription levels of *Pm*Spätzle (**A**), *Pm*MyD88 (**B**), *Pm*Dorsal (**C**) and *Pm*Relish (**D**). The data are shown as the mean ± standard deviation. An asterisk represents significant differences from control group (*p* < 0.05). The experiment was carried out in triplicate.

As shown in Fig. 3A,B, YHV infection significantly enhanced the transcription of *Pm*Spätzle and myeloid differentiation factor 88 (MyD88) in the Toll pathway. *Pm*Spätzle was increased by 12, 16, 7, 10, 10, 3 -fold at 6, 12, 18, 24, 30 and 36 hpi, respectively, while *Pm*MyD88 gradually increased and reached the highest level (sixfold) at 18 hpi. This suggested that the Toll signaling pathway responded to YHV infection. Notably, expression of *Pm*Dorsal in YHV-challenged shrimp remained at a similar level, compared with that in non-infected shrimp (Fig. 3C).

Next, the RNA interference experiment was carried out in order to investigate the influence of *Pm*AP2-β on signaling pathways and other immune-related genes during YHV infection. In previous research, we have shown that *Pm*AP2-β transcript can be efficiently suppressed by *Pm*AP2-β dsRNA^7^. Interestingly, expression of *Pm*Spätzle in *Pm*AP2-β silenced shrimp challenged with YHV was highly up-regulated by 31- and 33-fold at 6 and 12 hpi, compared with non-infected shrimp (Fig. 3A). In addition, *Pm*AP2-β silencing increased the expression of *Pm*MyD88 at 6, 18, 30 and 36 hpi (Fig. 3B), as well as *Pm*Dorsal at 6 and 12 hpi (Fig. 3C). Clearly, *Pm*AP2-β mediates the Toll signaling pathway during YHV infection.

On the contrary, YHV infection only induced the expression of *Pm*Relish, representing the Imd pathway, by threefold at 18 hpi, and *Pm*AP2-β silenced shrimp did not show significant changes in *Pm*Relish expression during YHV infection, except at 12 hpi (Fig. 3D). It is likely that the Imd pathway may not play an essential role in response to YHV infection.

**Figure 4:**
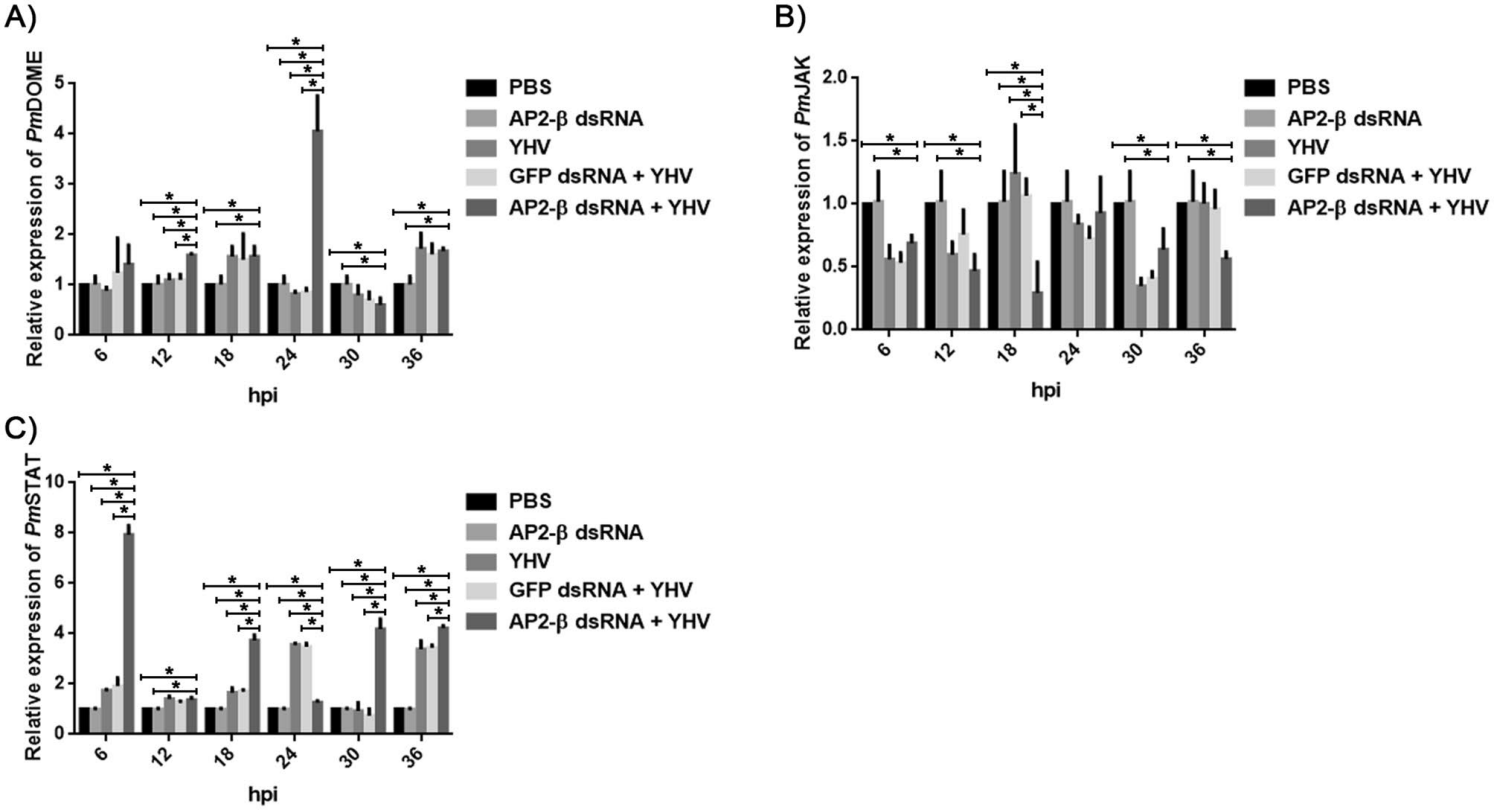
Effect of *Pm*AP2-β silencing on the JAK/STAT pathways during YHV infection. *Pm*AP2-β knockdown was carried out as described in "Methods". The transcription levels of *Pm*DOME (**A**), *Pm*JAK (**B**) and *Pm*STAT (**C**) at 6, 12, 18, 24, 30, 36 h after YHV infection were determined by qRT-PCR. The data are shown as the mean ± standard deviation. An asterisk represents significant differences from control group (*p* < 0.05). The experiment was carried out in triplicate.

In addition, the role of the JAK/STAT signaling pathway during YHV infection was investigated by measuring the transcription levels of *Pm*DOME, *Pm*JAK and *Pm*STAT. Figure 4A showed that YHV-challenged shrimp have a similar expression of *Pm*DOME, compared with that in non-infected shrimp. Meanwhile, *Pm*JAK was mostly down-regulated during YHV infection (Fig. 4B), while *Pm*STAT expression remained unchanged upon YHV infection, except at 24 and 36 hpi, at which *Pm*STAT was up-regulated around threefold (Fig. 4C). Silencing of *Pm*AP2-β increased expression of *Pm*DOME in YHV-challenged shrimp by fourfold at 24 hpi (Fig. 4A) and caused an up-regulation of *Pm*STAT by eightfold at 6 hpi and by approximately fourfold at 18, 30 and 36 hpi (Fig. 4C), in comparison with non-challenged shrimp. This result indicated that *Pm*AP2-β might be associated with *Pm*STAT activation.

### Effect of *Pm*AP2-β silencing on the expression of antimicrobial peptides during YHV infection

**Figure 5:**
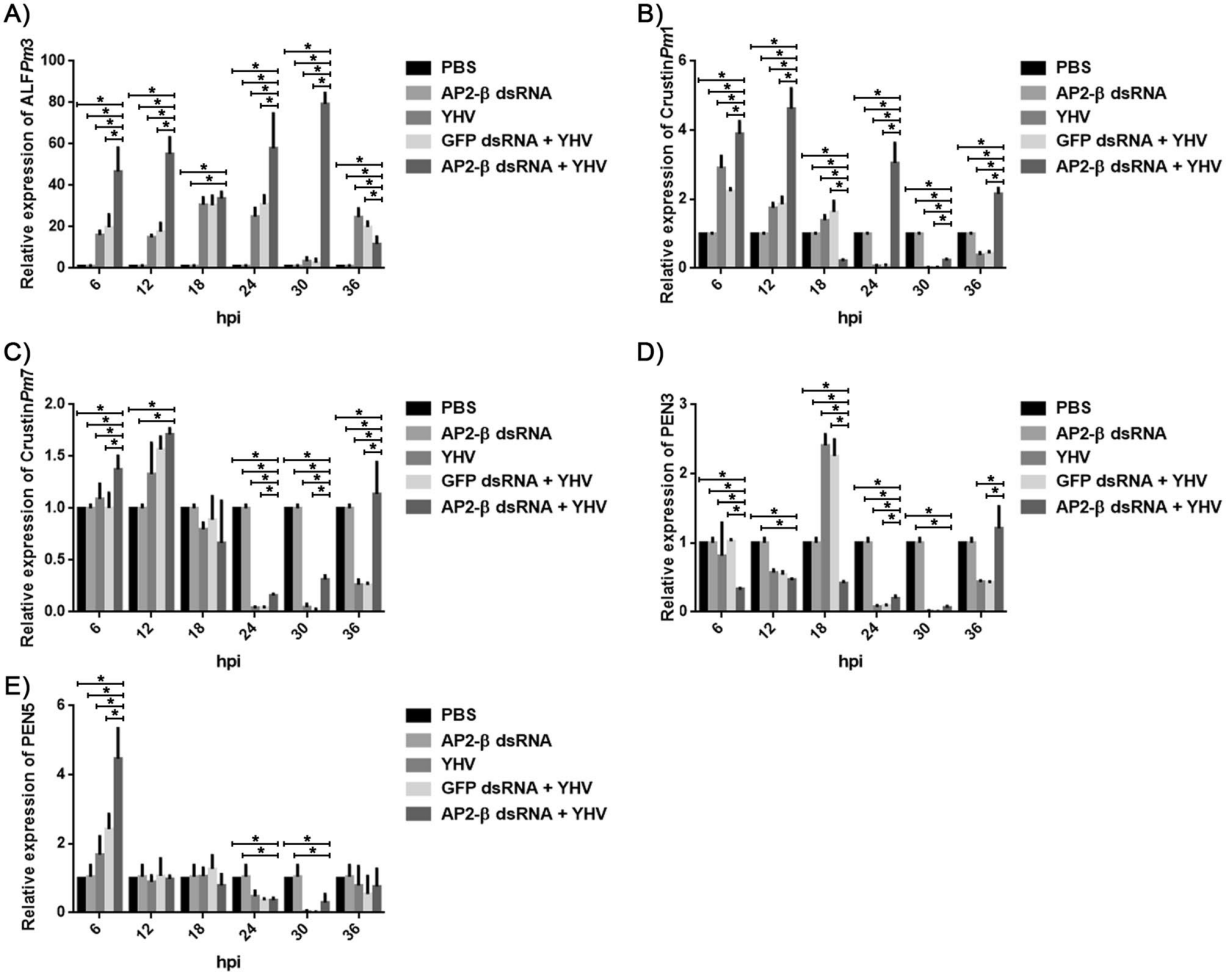
Determination of the mRNA transcription levels of antimicrobial peptides under influence of *Pm*AP2-β knockdown during YHV infection. ALF*Pm*3 (**A**), Crustin*Pm*1 **(B**), Crustin*Pm*7 (**C**), PEN3 (**D**) and PEN5 (**E**) transcripts of *Pm*AP2-β-silenced shrimp challenged by YHV at 6, 12, 18, 24, 30, 36 h were quantified by qRT-PCR and compared with those in unchallenged *Pm*AP2-β-silenced, YHV-challenged, and YHV-challenged + GFP knockdown shrimp.

In this work, we investigated the influence of *Pm*AP2-β knockdown on the expression of ALF*Pm*3, Crustin*Pm*1, Crustin*Pm*7, PEN3 and PEN5. Figure 5A showed that ALF*Pm*3 was highly up-regulated by 16, 15, 30, 24, 3, and 25-fold at 6, 12, 18, 24, 30 and 36 h after YHV infection, respectively. Crutin*Pm*1 was increased by threefold at 6 h upon YHV infection (Fig. 5B), while PEN3 was up-regulated at the highest level at 18 hpi (Fig. 5D). In contrast, Crustin*Pm*7 and PEN5 seemed to give minimal response to YHV infection (Fig. 5C,E).

Silencing of *Pm*AP2-β significantly increased ALF*Pm*3 transcripts by 47, 55, 34, 58, 79, 12-fold at 6, 12, 18, 24, 30 and 36 hpi (Fig. 5A). Similarly, knockdown of *Pm*AP2-β enhanced Crutin*Pm*1 transcription level by 4, 5 and threefold at 6, 12 and 24 h post-YHV infection (Fig. 5B). It is worth noting that expression of PEN5 in *Pm*AP2-β silenced shrimp was also increased by 4.5-fold at 6 h after YHV challenge (Fig. 5E), while *Pm*AP2-β silencing did not enhance the expression of Crustin*Pm*7 and PEN3 (< twofold) (Fig. 5C,D). Clearly, ALF*Pm*3 and Crutin*Pm*1 play an important role during YHV infection and their expressions were influenced by *Pm*AP2-β.

### ALF*Pm*3 and crustin*Pm*1 are responsible for defence against YHV

**Figure 6:**
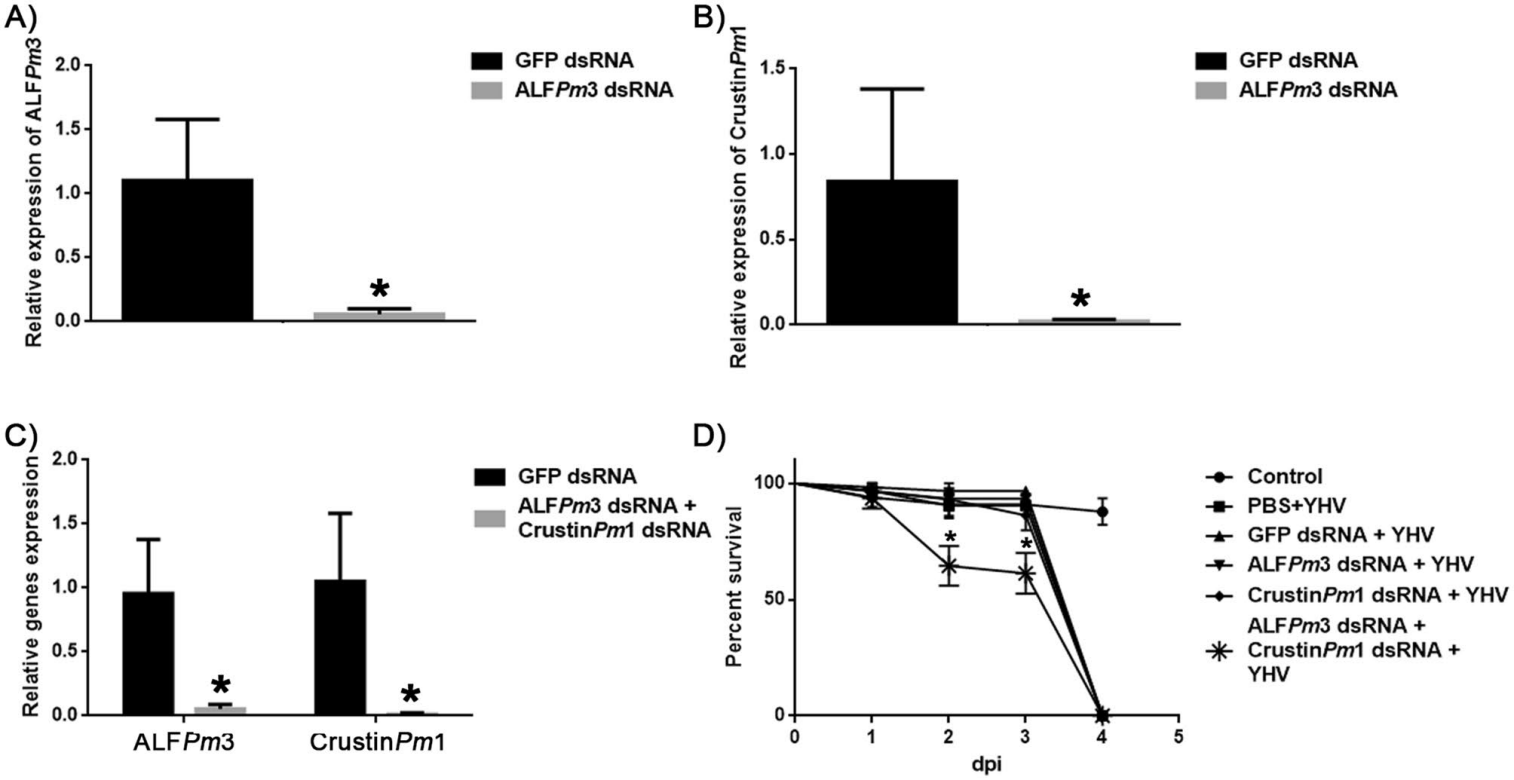
Effect of ALF*Pm*3 and Crustin*Pm*1 knockdown during YHV infection. Determination of ALF*Pm*3 and Crustin*Pm*1 transcript level in *P. monodon* hemocytes after shrimp were double injected with either 1 µg of ALF*Pm*3 dsRNA per 1 g shrimp (**A**) or 1 µg of Crustin*Pm*1 dsRNA per 1 g shrimp (**B**) or both (**C**). (**D**) Percent survival of ALF*Pm*3 or Crustin*Pm*1 knockdown shrimp upon YHV infection. Shrimp were divided into 6 groups, including PBS (control), YHV-challenged, YHV-challenged + GFP knockdown, YHV-challenged + ALF*Pm*3 knockdown, YHV-challenged + Crustin*Pm*1 knockdown and YHV-challenged + ALF*Pm*3/Crustin*Pm*1 knockdown. Each group contained 10 shrimps and the experiment was carried out in triplicate.

Roles of ALF*Pm*3 and Crustin*Pm*1 against YHV were further investigated. Either ALF*Pm*3 or Crustin*Pm*1 or both ALF*Pm*3 and Crustin*Pm*1 were knocked down using ALF*Pm*3 dsRNA and/or Crustin*Pm*1 dsRNA of 1 µg per 1 g shrimp as described in “Methods”. Figure 6A–C showed that ALF*Pm*3 and Crustin*Pm*1 were successfully knocked down. Silencing of either ALF*Pm*3 or Crustin*Pm*1 alone did not alter shrimp’s survival rate upon YHV infection (Fig. 6D). However, knockdown of both ALF*Pm*3 or Crustin*Pm*1 significantly reduced survival percentage at day 2 and 3 post-YHV infection. It is likely that ALF*Pm*3 and Crustin*Pm*1 covered for each other in a defence against YHV.

## Discussion

Clathrin-mediated endocytosis plays an essential role in YHV entry into shrimp cells^4,5^. In this work, we studied the effects of *Pm*AP2-β silencing on gene expression and shrimp mortality during YHV infection. *Pm*AP2-β is a large subunit 2β of the AP-2 complex, which interacts with clathrin. Previously, *Pm*AP2-β has been characterized and was shown to play a role during WSSV infection^7^.

In this work, *Pm*AP2-β was continuously up-regulated more than twofold during YHV infection (Fig. 1A). In addition, immunofluorescence showed that the level of *Pm*AP2-β protein was also increased in YHV-challenged hemocyte cells, compared with non-infected cells (Fig. 1B). Figure 2 illustrated that clusters of *Pm*AP2-β located at the plasma membrane of YHV-infected shrimp cells and the sac structures of *Pm*AP2-β, found in the cytoplasm, may contain the virus inside. Knockdown of *Pm*AP2-β gave rise to a delay of shrimp mortality (Fig. 1C), as well as a reduction in YHV copy number (Fig. 1D). Clearly, silencing of *Pm*AP2-β disrupted YHV propagation. This may be a result of lower number of YHV entering shrimp cells via clathrin-mediated endocytosis or the silencing of *Pm*AP2-β triggering shrimp immune responses.

In *Drosophila*, Spätzle has been characterized as the cytokine-like molecule that binds to Toll receptor, resulting in signaling cascade through MyD88 and transcription factor Dorsal^29,30^. In this work, the transcription of *Pm*Spätzle and *Pm*MyD88 was up-regulated during YHV infection (Fig. 3A,B), suggesting that YHV activated the Toll pathway. Silencing *Pm*AP2-β dramatically increased *Pm*Spätzle by 31- and 33-fold at 6 and 12 h after YHV challenge (Fig. 3A) and also enhanced *Pm*MyD88 and *Pm*Dorsal expression levels (Fig. 3B,C). In unchallenged shrimp, *Pm*AP2-β knockdown did not affect *Pm*Spätzle, *Pm*MyD88 and *Pm*Dorsal expression, however, *Pm*AP2-β silenced shrimp exhibited significantly higher expression of these genes during YHV infection, compared with YHV-challenged normal shrimp. This indicated that *Pm*AP2-β may have an influence on the Toll pathway during YHV infection.

Depletion of *Pm*AP2-β seemed to amplify cellular response of the Toll signaling pathway toward YHV infection. In general, endocytosis mediates receptor signaling by (1) controlling the number of receptors present on the plasma membrane for binding extracellular ligands (2) degradation or recycling of internalized receptors modulates the strength and specificity of signal transmission (3) endosomes play a part in intracellular signaling^31–33^. During *Drosophila* embryogenesis, Toll signaling was suggested to occur from the endosome rather than on the plasma membrane^34^. In *P. monodon*, silencing of early endosome antigen 1 (EEA1) protein (*Pm*EEA1), involving in early endosome fusion, caused a delay in shrimp mortality due to YHV infection^35^. Similar results were observed in YHV-challenged shrimp with either *Pm*Rab7 or *Pm*Rab11 suppression^6,36^. These suggested that endosome trafficking plays an important role during YHV infection. It is possible that lack of *Pm*AP2-β may impair clathrin-mediated endocytosis, resulting in alteration of signaling. It was previously reported that clathrin and dynamin-deficient cells showed enhanced activation of canonical NF-κB signaling^25^.

Regarding the Imd signaling pathway, *Pm*Relish expression did not increase significantly during YHV infection and *Pm*AP2-β-deficiency seemed not to influence *Pm*Relish transcript (Fig. 3D). This implied that the Imd pathway may not substantially contribute to YHV infection and *Pm*AP2-β deficiency did not affect the Imd signaling. Somehow, it was previously reported that *Pm*Relish silencing made the shrimp more susceptible to YHV^37^. In *Drosophila*, the Imd pathway regulates immune genes against Gram-negative bacteria^38^ and also possesses antiviral function^39,40^. In Chinese white shrimp *Fenneropenaeus chinensis*, *FcIMD* was up-regulated upon WSSV challenge, suggesting that the Imd signaling pathway was involved in antiviral innate immunity of shrimp. It was reported that knockdown of Relish affected the activity of phenoloxidase (PO) and superoxide dismutase (SOD), and total hemocyte count (THC) after WSSV or *Vibrio alginolyticus* infection in crab *Scylla paramamosain*^41^.

Based on *Pm*DOME, *Pm*JAK and *Pm*STAT expression, the JAK/STAT did not promptly respond to YHV infection at an early stage, when only *Pm*STAT was up-regulated around threefold at 24 and 36 hpi (Fig. 4). However, under the suppression of *Pm*AP2-β, the *Pm*STAT transcript significantly increased by eightfold at 6 hpi and by fourfold at 18, 30 and 36 hpi, in response to YHV (Fig. 4C). Devergne and colleagues reported that, in *Drosophila*, recruitment and trafficking of the clathrin-AP complexes into endocytic vesicles towards the lysosome could enhance the JAK/STAT signaling^21^. In contrast, Vidal and co-workers suggested that endocytic trafficking acts as a negative regulator of JAK/STAT signaling in *Drosophila*^42^. We postulated that knockdown of *Pm*AP2-β may disrupt clathrin-dependent endocytosis and signaling from endocytic mechanisms, resulting in an increased expression of *Pm*STAT. It is possible that in *P. monodon*, endocytic mechanisms modulate the JAK/STAT signaling negatively.

Regulation of signaling pathways could alter the expression level of antimicrobial peptides (AMPs). In Kuruma shrimp *Marsupenaeus japonicus*, Gram-positive and Gram-negative bacteria can activate the Toll pathway by their pathogen-associated molecular patterns (PAMPs) directly binding to Toll-like receptors, enhancing the expression of AMPs such as ALF-B1, ALF-C2, CruI-1 and CruI-3^43^. Furthermore, injection of activated *Pm*Spätzle enhanced transcription levels of ALF*Pm*3, crustin*Pm*1, crustin*Pm*7 and penaeidin3 in black tiger shrimp^44^. The recombinant Spätzle-like protein from Chinese shrimp, *Fenneropenaeus chinensis* could also increase crustin 2 expression in crayfish^45^.

Silencing of *Pm*Relish shrimp suppressed the expression level of penaeidin5, but did not affect ALF*Pm*3, crustin*Pm*1 and penaeidin3 expression levels^37^. Knockdown of *IMD* in *Procambarus clarkii* inhibited the expression of *Cru1* and *2*, *ALF 1* and *2* and *Lys1* in red swamp crayfish challenged with *Vibrio anguillarum*^46^. In crab *S. paramampsain*, Relish knockdown caused a downregulation of immune genes such as JAK, crustin and prophenoloxidase^41^.

Regarding the JAK/STAT, knockdown of suppressor of cytokine signaling 2 (SOCS2) increased ALF-C1, C2 and D1, and Crustin I expression levels upon *V. anguillarum* challenge^47^. Meanwhile, injection of recombinant SOCS2 reduced STAT phosphorylation and inhibited STAT translocation into the nucleus, resulting in a decline in the AMP expression.

Since *Pm*AP2-β seemed to regulate the signaling cascades, effects of *Pm*AP2-β silencing on AMP expression have been investigated. In general, ALFs showed broad antimicrobial activity against Gram-positive and Gram-negative bacteria, fungi and viruses, while crustins mainly exhibited antibacterial activity and penaeidins mostly functioned against bacteria and fungi. Previously, a suppression subtractive hybridization (SSH) study reported apparently up-regulated AMPs, including ALF*Pm*6 and crustin*Pm*1 in response to YHV infection^48^. Figure 5 showed that among five AMPs (ALF*Pm*3, Crustin*Pm*1, Crustin*Pm*3, PEN3 and PEN5), ALF*Pm*3 was the most active AMPs against YHV. *Pm*AP2-β depleted shrimp showed a significant increase in both ALF*Pm*3 and Crustin*Pm*1 expressions during YHV infection, compared with normal shrimp challenged with YHV. We postulated that *Pm*AP2-β depletion amplified the Toll signaling during YHV infection, resulting in elevated levels of ALF*Pm*3 and Crustin*Pm*1. Consistent with this, *Pm*AP2-β-deprived shrimp were more resistant to YHV, than normal shrimp (Fig. 2C). In addition, ALF*Pm*3 and Crustin*Pm*1 silenced shrimp had lower survival rate on days 2 and 3, compared with normal shrimp infected by YHV (Fig. 6D). This indicated that ALF*Pm*3 and Crustin*Pm*1 are important in defence against YHV. Previous research demonstrated that crustin*Pm*1 was found in the granule-containing hemocytes targeted by YHV^49^.

In conclusion, this research suggested that clathrin-mediated endocytosis not only functions as an entry route for YHV but also plays a role in regulating the intracellular signals. *Pm*AP2-β depletion stimulated the Toll signaling, resulting in elevated levels of ALF*Pm*3 and Crustin*Pm*1 during YHV infection. Both ALF*Pm*3 and Crustin*Pm*1 are essential antimicrobial peptides, acting against YHV.

## Methods

### Shrimp

Healthy black tiger shrimp, *P. monodon*, of about 3.23 ± 0.15 g bodyweight, were from Charoen Pokphand Farm in Chanthaburi Province, Thailand. They were acclimated in laboratory tanks (120 L) at ambient temperature (**28 ± 4** °C) and maintained in aerated water with a salinity of **20** ppt for at least 1 week before starting the experiments.

### YHV stock preparation

YHV stock was prepared as described in previous study^4^. Briefly, hemolymp was drained from YHV-infected moribund shrimp by 1 ml syringe containing an equal volume of modified Alsever solution (MAS: 27 mM sodium citrate, 336 mM NaCl, 115 mM glucose, 9 mM EDTA, pH 7.0). Hemocytes were removed by centrifugation at 1000× *g* for 10 min at 4 °C. The supernatant was filtered with 0.45 µm MILLEX-HP filter unit and centrifuged at 30,000× *g* for 30 min at 4 °C. The pellet was washed twice with TN buffer (50 mM Tris–HCl, pH 7.4 and 100 mM NaCl), then, aliquoted and kept at − 80 °C until use. YHV copy number was quantified by qRT-PCR using a specific primer pair for YHV genome (YHV-141-F and YHV-206-R in Table S1)^50^.

### Expression of *Pm*AP2-β during YHV infection

Healthy shrimp were separated into two groups, each of which consists of nine individuals, and was injected with either 50 µl of PBS or YHV (500,000 copies). Hemolymph was withdrawn from the abdomen connecting to the first pleopod using a 26-gauge needle and a 1 ml syringe containing an equal volume of ice-cold MAS solution. Each sample contains hemolymph from 3 shrimps (approximately 200 µl of hemolymph per individual). Hemocytes were pelleted by centrifugation at 800× *g* for 10 min at 4 °C. Total RNA was extracted by FavorPrep Tissue Total RNA mini kit (Favogen) and followed by cDNA synthesis using RevertAid First Strand cDNA Synthesis kit (ThermoFisher). *Pm*AP2-β transcription level was quantified by qRT-PCR using specific primers for *Pm*AP2-β (Supplementary Information, Table S1). Elongation factor-1 alpha (EF-1α) gene was used as an internal control. The experiment was performed in triplicate. Mathematical model was used to analyze the threshold cycle (C_T_)^51^. Statistical analysis was done using the one-way ANOVA followed by a post hoc test. The result differences were considered significant at *p* < 0.05 (*).

Comparative *C*_T_ method was employed to compare the gene expression in two different samples. The fold change of gene expression was calculated as follows:

$${\text{Fold}}\;{\text{change}} = 2^{{ - \Delta \Delta {\text{CT}}}}$$

$$\begin{aligned} \Delta \Delta C_{T} & = \left[ {\left( {C_{{\text{T}}} \;{\text{gene}}\;{\text{of}}\;{\text{interest }} - C_{{\text{T}}} \;{\text{internal}}\;{\text{control}}} \right)\;{\text{sample}}\;{\text{A}}} \right. \\ & \quad \left. { - \left( {C_{{\text{T}}} \;{\text{gene}}\;{\text{of}}\;{\text{interest}} - C_{{\text{T}}} \;{\text{internal}}\;{\text{control}}} \right)\;{\text{sample}}\;{\text{B}}} \right] \\ \end{aligned}$$

### Immunofluorescence confocal microscopy

Either diluted YHV stock solution (approximately 10,000 copies per µl) or 150 mM NaCl was injected into shrimp. The hemolymph was collected at 24 h post-injection and mixed in an equal volume of 4% paraformaldehyde in PBS. Hemocytes were collected by centrifugation (800× *g* for 10 min at 4 °C), washed 3 times with PBS and fixed on microscope slides. Hemocytes were incubated with 0.1% Triton X-100 in PBS for 5 min and washed 3 times with PBS. Purified rabbit anti-AP2-β (Abcam) polyclonal IgG antibody in 1:50 dilution in PBSF (PBS with 1% (v/v) FBS) was used to probe *Pm*AP2-β, followed by Alexa Fluor 488 goat anti-rabbit IgG antibody (Invitrogen), diluted 1:500. YHV was detected by monoclonal IgG antibody specific to gp116^52^, diluted 1:50 in PBSF, followed by a 1:1000 dilution of Alexa Fluor 568 goat anti-mouse IgG antibody (Invitrogen). Nuclei were stained with 1:1,000 dilution of Hoechst (ThermoFisher) in PBS. The microscope slides containing the stained and fixed hemocytes were then coated by ProLong Gold (Invitrogen) and kept in the dark at 4 °C until they were observed by a confocal fluorescence microscopy.

### Visualization of *Pm*AP2-β by TEM

Shrimp (3–5 g) were injected by YHV of approximately 10,000 copy numbers and gill tissues were then collected at 30 hpi and immediately fixed by 4% paraformaldehyde. Fixed tissues were then washed three times by ice-cold PBS and followed by the manufacturer’s protocol for embedding by LR White Embedding Medium (EMS). The embedded gills were cut into ultrathin sections (60–70 nm) and placed on a Formvar-supported nickel grid. The grids were incubated with 5% BSA in PBS for 1 h. A 10 nm gold particle was conjugated to primary AP-2β antibody (Abcam) using InnovaCoat Gold Conjugation kit. The gold conjugated antibody was diluted 1:50 by 1% BSA in PBS. The grids were incubated with diluted gold conjugated antibody solution at 4 °C overnight and stained with uranyl acetate solution for 5 min, followed by Reynolds lead citrate solutions for 2 min, and observed using Transmission Electron Microscope Libra 120 Plus (ZEISS) at the Microscopy Unit of IBT-UNAM.

### Mortality assay of *Pm*AP2-β silencing shrimp upon YHV infection

Double-strand RNA of *Pm*AP2-β and GFP were prepared as described previously^7^. In brief, the PCR products (*Pm*AP2-β and GFP) were amplified separately by specific primers (Supplementary Information, Table S1) with the following conditions: 94 °C for 3 min (denaturation), followed by 40 cycles of 94 °C for 30 s, 55 °C for 30 s and 72 °C for 30 s and a final extension at 72 °C for 10 min. The two PCR product templates were in vitro transcribed using the T7 RiboMAX System (Promega) to produce two complementary single-stranded RNAs. Then, RQ1 RNase- free DNase was added and incubated at 37 °C for 1 h and the single-stranded RNAs were then purified by standard phenol–chloroform extraction. To generate dsRNA, equal amounts of each of the complementary single-stranded RNAs were mixed, incubated at 70 °C for 10 min, and slowly cooled down at room temperature. The quality and quantity of *Pm*AP2-β dsRNA and GFP dsRNA were analyzed by 1% agarose gel electrophoresis and absorbance at 260 nm, respectively.

To study the effect of *Pm*AP2-β silencing, black tiger shrimp were divided into four groups, each of which consisted of 10 individuals and were injected with either PBS (group 1, control), PBS + YHV (group 2), 10 µg of *Pm*AP2-β dsRNA per 1 g of shrimp + YHV (group 3) and 10 µg of GFP dsRNA per 1 g of shrimp + YHV (group 4). In this experiment, a diluted YHV solution containing approximately 10,000 copies per µl was injected into shrimp at 24 h after PBS or dsRNA injection. The mortality was recorded every 12 hpi up to 8 days. This experiment was carried out in triplicate. Data were analyzed using GraphPad Prism 6 plot, and presented as percent survival with the *p* values calculated by log-rank test.

### Influence of *Pm*AP2-β silencing on YHV copy numbers and *P. monodon* immune related genes during YHV infection

Shrimp hemocytes were collected at different timepoints (6, 12, 18, 24, 30 and 36 h-post injection) from *P. monodon* treated with YHV, GFP dsRNA + YHV and *Pm*AP2-β dsRNA + YHV. Total RNA extraction and cDNA synthesis were performed as described previously. *P. monodon* immune related genes and YHV copy number were quantified by quantitative RT-PCR using specific primers as shown in Supplementary Information, Table S1. Elongation factor-1 alpha (EF-1α) gene was used as an internal control. The experiment was performed in triplicate and the mathematical model was used to analyze the threshold cycle (C_T_). Statistical analysis was done using the one-way ANOVA followed by a post hoc test. The result differences were considered significant at *p* < 0.05 (*).

### Mortality assay of ALF*Pm*3 and/or Crustin*Pm*1 knockdown shrimp upon YHV infection

The DNA amplicon templates of ALF*Pm*3 and Crustin*Pm*1 were amplified using primers in Supplementary Information, Table S1 and ALF*Pm*3 and Crustin*Pm*1 dsRNA synthesis was performed as described above. Either ALF*Pm*3 dsRNA or Crustin*Pm*1 dsRNA was injected at 1 µg per 1 g of shrimp and the hemocytes were collected at 24 hpi. Total RNA and cDNA synthesis were performed as described above; and the level of ALF*Pm*3 and Crustin*Pm*1 transcripts were determined by qRT-PCR. In the mortality experiment, shrimp were divided into 6 groups with 10 shrimps per group as followed, Group 1: PBS (control), Group 2: YHV-challenged, Group 3: YHV-challenged + GFP dsRNA, Group 4: YHV-challenged + ALF*Pm*3 dsRNA, Group 5: YHV-challenged + Crustin*Pm*1 dsRNA, and Group 6: YHV-challenged + ALF*Pm*3/Crustin*Pm*1 dsRNAs. After YHV injection, shrimp mortality was recorded every 12 h up to 4 days. The experiment was performed in triplicate and the data were analyzed using GraphPad Prism 6 and presented as percent survival with the *p* values calculated by log-rank test.

## Supplementary Information

Supplementary Information.

## Abbreviations

ALFs: Anti-lipopolysaccharide factors
AMPs: Antimicrobial peptides
Imd: Immune deficiency
*Pm*: *Penaeus monodon*
WSSV: White spot syndrome virus
YHV: Yellow head virus
