# Supplementary material for: PmAP2-β depletion enhanced activation of the Toll signaling pathway during yellow head virus infection in the black tiger shrimp Penaeus monodon
Source: Sci Rep. 2021 May 18;11:10534. doi: 10.1038/s41598-021-89922-w (PMC8131699; doi:10.1038/s41598-021-89922-w)
Supplement: Supplementary file 1 — Supplementary Information. [file 41598_2021_89922_MOESM1_ESM.docx]

**Supplementary Information**

*Pm*AP2-β depletion enhanced activation of the Toll signaling pathway during yellow head virus infection in the black tiger shrimp *Penaeus monodon*

Thapanan Jatuyosporn ^1,2^_,_ Pasunee Laohawutthichai ^1,2^_,_ Premruethai Supungul ^3^, Rogerio R. Sotelo-Mundo ^4^, Adrian Ochoa-Leyva ^5^, Anchalee Tassanakajon ^2^ and Kuakarun Krusong ^1^

^1^ Structural and Computational Biology Research Unit, Department of Biochemistry, Faculty of Science, Chulalongkorn University, Bangkok 10330, Thailand

^2^ Center of Excellence for Molecular Biology and Genomics of Shrimp, Department of Biochemistry, Faculty of Science, Chulalongkorn University, Bangkok 10330, Thailand

^3^ National Center for Genetic Engineering and Biotechnology (BIOTEC),

National Science and Technology Development Agency (NSTDA), Pathumthani 12120, Thailand

^4^ Laboratorio de Estructura Biomolecular, Centro de Investigación en Alimentación y Desarrollo, A.C. (CIAD). Carretera Gustavo Enrique Astiazaran Rosas No. 46, Hermosillo, Sonora 83304, Mexico

^5^ Departamentos de Microbiología Molecular, Universidad Nacional Autónoma de México (UNAM), Avenida Universidad 2001, Colonia Chamilpa, Cuernavaca 62210, Mexico

* To whom correspondence should be addressed:

Kuakarun Krusong, Ph.D.

Department of Biochemistry, Faculty of Science, Chulalongkorn University, Bangkok 10330, Thailand, Tel: +66 (0)2 218 5413, Email: Kuakarun.K@chula.ac.th

**Table S1** Nucleotide sequences of the primers

| **Primer name** | **Sequence (5’ – 3’)** | **Experiment** |
| --- | --- | --- |
| knAP-2bT7-F | GGATCCTAATACGACTCACTATAGG CAACCAGCAGCAAGCACCACAG | double-stranded RNA synthesis |
| knAP-2bT7-R | GGATCCTAATACGACTCACTATAGG TGGTGTGCTTGAGGAGCCAATG | double-stranded RNA synthesis |
| knAP-2b-F | CAACCAGCAGCAAGCACCACAG | double-stranded RNA synthesis |
| knAP-2b-R | TGGTGTGCTTGAGGAGCCAATG | double-stranded RNA synthesis |
| knGFPT7-F | GGATCCTAATACGACTCACTATAGG ATGGTGAGCAAGGGCGAGGA | double-stranded RNA synthesis |
| knGFPT7-R | GGATCCTAATACGACTCACTATAGG TTACTTGTACAGCTCGTCCA | double-stranded RNA synthesis |
| knGFP-F | ATGGTGAGCAAGGGCGAGGA | double-stranded RNA synthesis |
| knGFP-R | TTACTTGTACAGCTCGTCCA | double-stranded RNA synthesis |
| knALF*Pm*3T7-F | TAATACGACTCACTATAGGTGTGTCCGTGCTGGTAAG | double-stranded RNA synthesis |
| knALF*Pm*3T7-R | TAATACGACTCACTATAGGTAAGGCTTGACGGTGAAC | double-stranded RNA synthesis |
| knALF*Pm*3-F | TGTGTCCGTGCTGGTAAG | double-stranded RNA synthesis |
| knALF*Pm*3-R | TAAGGCTTGACGGTGAAC | double-stranded RNA synthesis |
| knCrustin*Pm*1T7-F | TAATACGACTCACTATAGGCGCCTCCTGTAGGCGCTGGTG | double-stranded RNA synthesis |
| knCrustin*Pm*1T7-R | TAATACGACTCACTATAGGCAAGAAAACGTACCGGTGGGCAGGTG | double-stranded RNA synthesis |
| knCrustin*Pm*1-F | CGCCTCCTGTAGGCGCTGGTG | double-stranded RNA synthesis |
| knCrustin*Pm*1-R | CAAGAAAACGTACCGGTGGGCAGGTG | double-stranded RNA synthesis |
| EF1-α-F | GGTGCTGGACAAGCTGAAGGC | real-time RT-PCR |
| EF1-α-R | CGTTCCGGTGATCATGTTCTTGATG | real-time RT-PCR |
| *Pm*AP-2b-qRT-F | TCAAGAACAGCGTGGATGTG | real-time RT-PCR |
| *Pm*AP-2b-qRT-R | AATACCATCCGCATTCAGATTG | real-time RT-PCR |
| *Pm*STAT-qRT-F | TATATCCGAATGTGCCTAAG | real-time RT-PCR |
| *Pm*STAT-qRT-R | ATAGTTTGTGGTGTGTTGGG | real-time RT-PCR |
| *Pm*Spätzle-qRT-F | TAAGCAAGGAGCAGGAAGAG | real-time RT-PCR |
| *Pm*Spätzle-qRT-R | TGGCATACACCACATCTGAG | real-time RT-PCR |
| *Pm*Dorsal-qRT-F | TCACTGTTGACCCACCTTAC | real-time RT-PCR |
| *Pm*Dorsal-qRT-R | GGAAAGGGTCCACTCTAATC | real-time RT-PCR |
| *Pm*Relish-qRT-F | TCTCCAGGTGAGCACTCAGTTGGC | real-time RT-PCR |
| *Pm*Relish-qRT-R | GCTGTAGCTGTTGCTGTTGTTGAG | real-time RT-PCR |
| ALF*Pm*3-F | CCCACAGTGCCAGGCTCAA | real-time RT-PCR |
| ALF*Pm*3-R | TGCTGGCTTCTCCTCTGATG | real-time RT-PCR |
| *Pm*DOME-qRT-F | CTCAGGCTATGTTTCTCAGGATTCA | real-time RT-PCR |
| *Pm*DOME-qRT-R | CACGGCAGTTCCTTTATGGTCT | real-time RT-PCR |
| YHV-141-F | CGTCCCGGCAATTGTGAT | real-time RT-PCR |
| YHV-206-R | CCAGTGACGTTCGATGCAATA | real-time RT-PCR |
| *Pm*PEN3-F | GGCTTAGCCCCTTACA | real-time RT-PCR |
| *Pm*PEN3-R | GACCCATACCTACAAATAAC | real-time RT-PCR |
| *Pm*PEN5-F | ATCCCGACCTATTAGTACTC | real-time RT-PCR |
| *Pm*PEN5-R | TTATCCTTTCAATGCAGAACAA | real-time RT-PCR |
| Crustin*Pm*1-F | CTGCTGCGAGTCAAGGTATG | real-time RT-PCR |
| Crustin*Pm*1-R | AGGTACTGGCTGCTCTACTG | real-time RT-PCR |
| Crustin*Pm*7-F | GGCATGGTGGCGTTGTTCCT | real-time RT-PCR |
| Crustin*Pm*7-R | TGTCGGAGCCGAAGCAGTCA | real-time RT-PCR |
| *Pm*JAK-F | TGCTGTTCCGACTGCGTTTC | real-time RT-PCR |
| *Pm*JAK-R | GCGTGGAAGTCTGCTCGAAC | real-time RT-PCR |
| *Pm*MyD88-F | GTGCACCAGAGTCATTGTAG | real-time RT-PCR |
| *Pm*MyD88-R | GGGAGTGGCAGAAACTTATC | real-time RT-PCR |

The double-underline indicates the T7 promotor sequence.
